# Autonomous, bidding, credible, decentralized, ethical, and funded (ABCDEF) publishing

**DOI:** 10.12688/f1000research.130188.2

**Published:** 2023-10-05

**Authors:** Taiki Oka, Kaito Takashima, Kohei Ueda, Yuki Mori, Kyoshiro Sasaki, Hiro Taiyo Hamada, Masahito Yamagata, Yuki Yamada

**Affiliations:** 1 Clinical Psychology, Graduate School of Human Sciences, Osaka University, Suita, Japan; 2 The Department of Decoded Neurofeedback, Computational Neuroscience Laboratories, Advanced Telecommunications Research Institute International, Kyoto, Japan; 3 Department of Neuropsychiatry, Faculty of Life Sciences, Kumamoto University, Kumamoto, Japan; 4 Graduate School of Human-Environment Studies, Kyushu University, Fukuoka, Japan; 5 Faculty of Informatics, Kansai University, Takatsuki, Japan; 6 Research & Development Department, Araya Inc., Tokyo, Japan; 7 Center for Brain Science and Department of Molecular and Cellular Biology, Harvard University, Cambridge, USA; 8 Faculty of Arts and Science, Kyushu University, Fukuoka, Japan

**Keywords:** decentralized science, registered reports, reproducibility, scientific ecosystem, funding

## Abstract

Scientists write research articles, process ethics reviews, evaluate proposals and research, and seek funding. Several strategies have been proposed to optimize these operations and to decentralize access to research resources and opportunities. For instance, we previously proposed the trinity review method, combining registered reports with financing and research ethics assessments. However, previously proposed systems have a number of shortcomings, including how to implement them, e.g., who manages them, how incentives for reviewers are paid, etc. Various solutions have been proposed to address these issues, employing methods based on blockchain technologies, called “decentralized science (DeSci)”. Decentralized approaches that exploit these developments offer potentially profound improvements to the troubled scientific ecosystem. Here, we propose a system that integrates ethics reviews, peer reviews, and funding in a decentralized manner, based on Web3 technology. This new method, named ABCDEF publishing, would enhance the speed, fairness, and transparency of scientific research and publishing.

## Introduction

*“By mutual confidence and mutual aid, Great deeds are done, and great discoveries made.”—Homer, “The Iliad”*

Current scientific endeavors have become complicated and protracted. Science advances by sifting through numerous findings made by many individuals and organizations. However, the current ecosystem of science suffers from various problems. First, researchers spend great amounts of time writing grant proposals to obtain research funding rather than conducting scientific investigations (Herbert et al. 2013). Funding decisions rely heavily on citation metrics, such as researcher h-indexes. ^1^^,^^2^ Second, it is now standard for human and animal research to undergo ethics reviews before being initiated. However, these reviews are usually carried out behind closed doors by ethics committees composed of members selected by each institution. ^3^ Additionally, such reviews are often ill-suited to evaluate research methods because the committees do not include experts in pertinent fields. ^4^ Moreover, approved proposals must often be revised due to unforeseen changes in experimental methodology, necessitating reassessment by *post hoc* ethics or peer review. ^5^ This rework could be prevented if methodological expertise review is done alongside the ethical review. Furthermore, proceeding on the assumption of the current institutional ethical reviews impedes broadening the scope of science, considering that many researchers suffer from the lack of opportunities for institutional review (Ref. 6; Independent researchers seeking ethic...). Third, infrastructure to support reproducibility and transparency has not yet been fully developed, ^7^^–^^9^ although there are a plethora of efforts to advance open science, such as open access, open data/material/code, open review reports, open peer review, pre-registration, and registered reports systems. Finally, there is excessive centralization of authority, ^10^^,^^11^ and funding is often concentrated on limited numbers of scientists and publishers. ^12^^,^^13^

To address these issues, we previously proposed a new procedure in which three types of peer review (scientific peer review, ethics review, and research funding review) are executed simultaneously on the same document. ^14^ This time-saving method is promising because it could solve the transparency problems, ethics review problems, and grant acquisition problems mentioned above. Nevertheless, the proposed system still relies on volunteer work of reviewers and influential publishers. Contrary to Homer’s assertion, modern scientific endeavor is generally performed in an opaque, unidirectional, biased, and centralized system.

As an alternative to this traditional system, Decentralized Science (DeSci) activities or systems are gaining popularity. ^15^^,^^16^ Multiple scientific entities are beginning to apply Web3 technology, most notably blockchain, to obtain research funding and to publish results. ^17^ Web3 is a general term for distributed networks where users autonomously exchange information and communicate. These entities are often run by autonomous organizations of scientists, called decentralized autonomous organizations (DAOs). Within a DAO, there is a central organization such as a working group (WG), but decision-making is decentralized, based on Web3 technology. Several DAOs are attempting to address the aforementioned problems. ^18^^,^^19^ Those DAOs deploy a review system created on a public blockchain, *e.g.,* Ethereum, to tackle transparency and incentive problems that have impaired conventional peer review. ^18^^,^^20^ In order to promote ethical behavior and inclusiveness, DAO systems implement a gamification mechanism that allows entire communities to evaluate peer reviews and vote for the best ones. There are also several DAOs that provide open budgets or data, *e.g.*, VitaDAO, GenomesDAO. Such DAOs collect their budgets by distributing tokens as voting rights to determine which projects are to be funded. Open data are held in decentralized storage, *e.g.,* InterPlanetary File System: IPFS, without censorship, and data transactions can be kept in the blockchain as an open ledger. Practically, however, DeSci systems have potential drawbacks. For example, they could damage interests of current publishers. Nonetheless, we hope this emerging movement will solve presently unresolved problems, *e.g.,* payment of incentives to reviewers, stability of reproducibility. ^17^^,^^20^^,^^21^

Here, we propose a system that integrates ethics reviews, peer reviews, and funding in a decentralized manner, based on Web3 technology. This autonomous, transparent, decentralized system would help shape cutting-edge scientific research and boost scientific transparency, efficiency, ethics, and reproducibility. We have already established a decentralized community called MinDAO to realize such a system as a host.

## ABCDEF publishing

**Figure 1.**
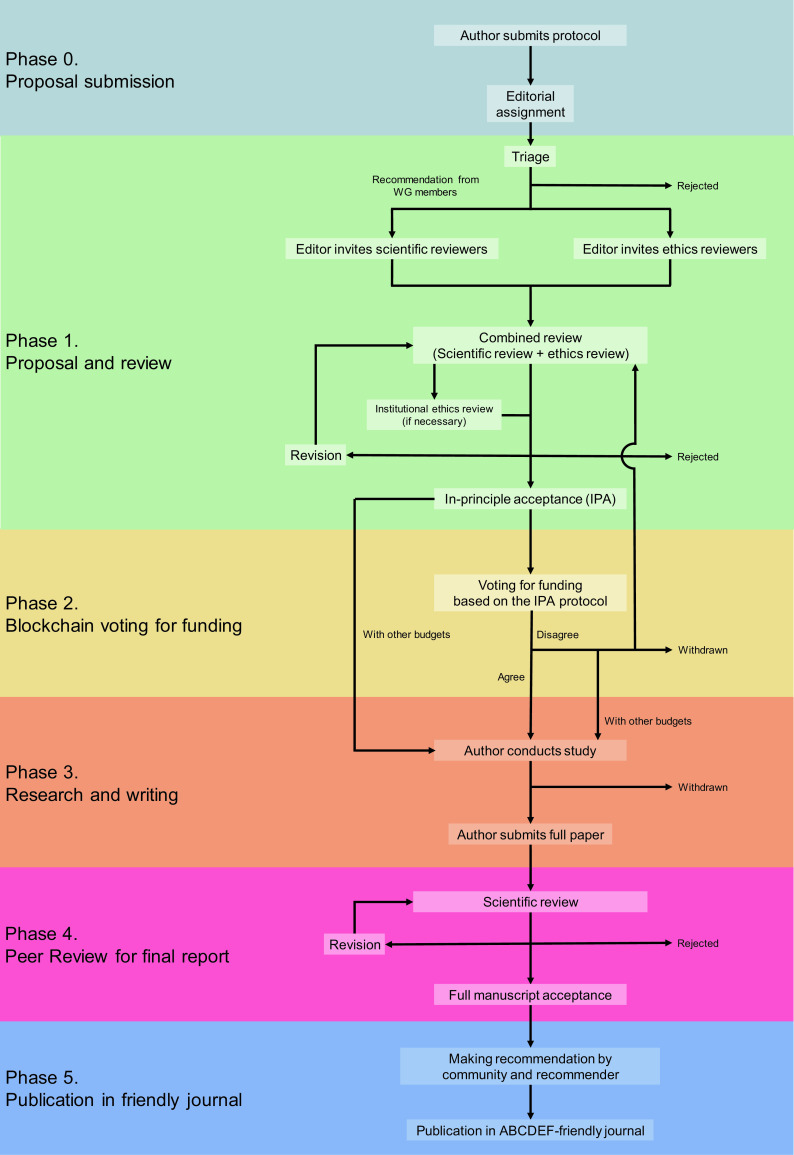
Flow of ABCDEF-publishing.

Figure 1 illustrates the scheme of a system we call “Autonomous, bidding, credible, decentralized, ethical, and funded (ABCDEF) publishing.” based on MinDAO. See the original concept described in our published article and its supplementary (Mori *et al.* (2022), ^14^ Supplementary ( https://osf.io/rq5vb)) for a detailed, phase-by-phase peer review scheme. After researchers write a research plan, there are five phases from reviewing to publishing. Here are how these phases would work.

### Phase 1. Proposal and review

A. *Scientific peer review and registered reports (RR)*

Each research proposal is peer-reviewed by multiple reviewers. In this process, peer reviewers evaluate the value of a research question, the rationale for the hypothesis, and the validity of methods for testing the hypothesis. RRs focus on the quality of methodology prior to data collection. Even in cases of exploratory research, peer reviewers evaluate scientific soundness, feasibility, and methodology of submitted proposals. ^22^

B. *Ethics reviews*

Several reviewers specializing in ethical analyses are assigned to each protocol to review ethics of the research. If reviewers have ethical concerns about a submitted protocol, authors must revise it in compliance with reviewer comments and judgments of editors. In cases in which ethical considerations not covered by the protocol are required, *e.g.,* clinical research, the protocol would be examined by author institutions.

Scientific reviewers assess the importance of research questions of a manuscript, the theoretical validity of proposed hypotheses, and whether experimental methods are appropriate for testing those hypotheses. Ethics reviewers assess the protocol for ethical issues. Scientific and ethics reviews are undertaken concurrently. Once the manuscript has successfully passed both reviews and is accepted in principle, authors can proceed to the funding review. If ethics reviewers determine that an institutional ethics review is necessary, an ethics review is conducted at author institutions. In this case, a funding review is possible only after passing institutional ethics reviews.

### Phase 2 Blockchain voting for funding

Once a protocol is accepted in Phase 1, voting for funding begins. The desired amount of funding is announced at the time of Phase 1 Peer Review. Voting on Phase 1 protocols occurs at this point, and funding is awarded from pooled funds. Community members evaluate Phase 1 protocols on their potential for scientific advancement and benefit to the public, and grants are made to protocols that meet these criteria. Voting is conducted primarily by community members possessing tokens. Although votes of individuals with more tokens have greater weight, they apply a voting system, such as quadratic funding, to minimize bias ^23^ (see the below details). Even if members disagree with funding for the protocol, researchers can proceed to the next phase using their own grants if they have them. Alternatively, researchers can revise their protocols and resubmit them for Phase 1 scientific review to be voted on again ( Figure 1). Also, DAO members who have the right to vote can require additional actions in both cases, *i.e.,* agreeing or disagreeing with comments by DAO members. If members disagree, but ask authors to revise their proposals, researchers can modify their protocols to obtain a grant.

In the traditional system, funding based on popularity and performance assessed with indicators such as the h-index is not necessarily a problem. However, funds are biased toward a small subset of researchers, and funding allocation may be skewed away from early-career researchers. To solve this issue, we will apply “quadratic funding,” limiting the percentage of famous and accomplished researchers receiving funds by route (√). ^23^ Quadratic Funding optimizes matching funds by prioritizing projects according to the number of voters. This ensures that funds are spent on projects that truly benefit the scientific community at large, not just a few prominent researchers.

Under this mechanism, the total amount of funding for a given issue is “the square root of each donation for that issue, *i.e.*, the square of the total amount”. Even small donations are strongly encouraged and funding democratically balances funds for the public good. After a successful funding vote, researchers can be supported by pooled funding sources. Participation in voting is restricted to credibility gained *via* activities ^24^ or Token holdings.

### Phase 3 Research and writing

This is the phase in which actual research and writing proceed, following the same flow as typical funded research. As described below, methods, experiments, and analyses conducted here are tied to the Phase 1 protocol in the blockchain. Therefore, deviations from or additions to the RR protocol and ethical review are explicitly indicated. Researchers conduct their studies following procedures accepted in Phase 1. Procedures are linked to the Phase 2 article using the blockchain. Therefore, if researchers deviate from their original protocol, this is apparent to readers.

### Phase 4 Peer review for the final report

This is a review resulting from protocols proposed and accepted in principle in Phase 1. Reviewers evaluate the quality of modifications and protocol deviations or ethical violations. In the event of rejection, after consultation, researchers would be asked to return the funds. Of course, peer reviewers should attempt to improve manuscripts by providing constructive discussions, as in traditional scientific peer review. Authors resubmit a Phase 2 article after data collection and analysis that contains the “Introduction” and “Methods” from the initial submission together with the “Results and Discussion” section. After the Phase 2 scientific evaluation is finished, the final manuscript will be published.

### Phase 5 Publishing in a friendly journal

The paper is published as a journal article. The community and the recommender make acceptance decisions as in Peer Community In (PCI) ( https://peercommunityin.org/), ^25^ and ABCDEF-friendly journals accept manuscripts selected, based on these recommendations.

Detailed handling of tokens and funds is described in Table 1. Through these five phases, transparency is ensured as the research process proceeds. Scientific reviewers and ethics reviewers would be invited by the community based upon license tokens, guaranteeing the quality of reviewers. Possible incentives for reviewers arising at each phase could include the following. Each reviewer/author would have a track record in the form of non-fungible tokens, which could be presented as credit for acquiring funding. ^26^ Incentives would be paid in tokens built on the blockchain. This incentive system is similar to that proposed in previous studies. ^18^^,^^20^ Researchers could also pay open access fees with tokens or other funds. Those grants would be provided from pooled funds. Funds would be handled by utilizing multiple funding systems, including donations or investments as external funding, and pooling funds with support from funding DAOs with quadratic funding and retrospective public goods funding. Another approach to grants involves journals of funding organizations initiating grant projects and allowing the submission of manuscripts to them. ^27^^,^^28^ There could also be a type of bounty for other community members *via* programmed, self-executing contracts, so-called smart contracts.

**Table 1.** Details of tokens and funding in each phase.

	When do tokens or money move?	Who obtains tokens or money?	What kind of tokens or money are used?
Phase 0 Submission of proposals	Assignment as reviewer	Anyone invited as reviewers	License token
Phase 1 Proposal and review	Peer-review completed	Reviewers	MinDAO token
Phase2 Blockchain voting for funding	Agreement to protocol	Authors	Money withdrawn in a pool of MinDAO for funds
Phase 3 Research and writing Phase 4 Peer Review for the final manuscript	Peer-review completed	Reviewers	MinDAO token
Phase 5 Publication in the friendly journal	Paying article processing charge (APC)	ABCDEF-friendly journal	MinDAO token or Money

## Advantages and opportunities

This section highlights advantages that this system would bring to the academic community. First, ABCDEF Publishing combines advantages of conventional review methods and blockchain-based techniques. It reduces the burden on researchers and provides incentives for all peer-review processes from the perspective of DeSci. Our past proposal integrated RRs, ethical reviews, and grant reviews. Also, as mentioned above, some previous articles have suggested incentivizing reviewers using blockchain-based tokens. ABCDEF Publishing would integrate all of these methods to improve efficiency, fairness, and transparency of the review process. Specifically, since a record of details of this review (who reviewed it and how, *etc.*) is kept on the blockchain or in decentralized storage, this authentication assurance is also important. This transparency enables funders to validate the security of their funding. Also, it maintains a record of how the budget was used. Use of funds can be documented. Furthermore, the blockchain would be able to link ethics statements and hypotheses that are registered in the RR system with methodologies and results. In addition, as seen in previous RRs, the ABCDEF system will accept submissions along with pilot data, replications of previous studies, and collections of various experiments and studies, allowing each content to be tied to the blockchain for reference. If that linkage is not made, it is possible to know that the method and analysis did not explicitly satisfy the registration.

Second, decentralized management would remove control from the hands of a few powerful administrators, leading to promotion of diverse research that contributes to science and more neutral evaluations of science and researchers. In addition, ABCDEF publishing would also benefit researchers whose institutions do not have ethics review boards and independent researchers who do not belong to specific institutions. ^29^^,^^30^ This system would enable such researchers to review their protocols from an ethics perspective quickly and easily. Although there is already an independent ethics review committee for such researchers, they must submit documents that are almost identical in content to those submitted for academic peer review in Stage 1 in their own format. Our proposal system could exclude the barrier by integrating all stages. Moreover, ABCDEF publishing would construct a reputational system based on activities of researchers/participants in a decentralized system of reviewers, independent of institutions and publishers (as in the Tenorio-Fornes proposal ^20^). Such a reputation system would allow independent researchers to be reviewed and to expand the review process range by themselves. The system of DeSci would also improve efficiency of all publishing procedures by decreasing office work using smart contract techniques, as previously proposed. ^20^

These advantages would allow ABCDEF-Publishing to achieve real distributed, citizen science. Nonetheless, ABCDEF Publishing does not seek to ignore or eliminate publishers, as has been claimed regarding the previously proposed DeSci system. ^18^ On the contrary, the credibility and reputation of publishers are critical and need to be supported. The previous proposal also recommended a review method using a DeSci system that is organized and transparent, while working with publishers. ^20^ We must respect the traditional scientific system that enables articles to be scientifically validated. Using ABCDEF Publishing, publishers could develop a more effective and efficient publishing system that would be mutually beneficial. Thus, it could help reform the complex systems of some journals, and indeed existing publishers are beginning to implement DeSci-based initiatives ( *e.g.*, https://twitter.com/ScisetsComm).

## Potential weaknesses, threats, and solutions

There are five potential pitfalls for ABCDEF Publishing.

1) It is still possible that a WG and some members of the DAO will hold too much power. Therefore, establishing a more democratic method of DAO management, depending on the number of non-fungible tokens (NFTs) owned, is crucial. The ideal is to employ a managerial system that is genuinely autonomous and decentralized.
2) As is often the case with token economics, tokens are unstable. Linking internal tokens ($MIN in our case) to current stablecoins is one option, *e.g.*, USDC, USDT. Stablecoins are digital assets focused on price stability. Due to price volatility brought on by the lack of underlying assets, crypto assets have not yet been used as a form of payment. Stablecoins were created to spread and boost the utility of these assets.
3) One concern is that fraudulent accounts, fabricated peer reviews, and fake contributions could appear. This issue is widely known as the Sybil attack, a type of attack on a computer service with many pseudonymous identities and enormous influence. Two types of defenses are proposed to thwart such abuses. One system is to evaluate risks of accounts using transaction data on the blockchain with machine learning. Another system uses non-exchangeable tokens linked to specific people, so-called soulbound tokens. ^31^^,^^32^ Moreover, it can lower the overall risk if they are tied to other accounts (e.g., ORCID, google accounts, github accounts). These systems allow us to predict risks of accounts and subtract the influence of potentially risky accounts. However, such proposals are still under development.
4) Another concern is about pairwise coordination of contributions in projects. For example, collaborators or unfriendly competitors work on the same project together in an attempt to control DAO decision-making. A similar idea in funding design is proposed to prevent such cases by controlling contributions based on social networks. ^33^ Another technique to minimize the contribution level to the cap has recently been used when users assist one another based on their shared activity history. ^34^
5) There may be instances in which these approaches fail, having various impacts, even on specific fields or where RRs are unnecessary, as in the humanities. Thus, a flexible flow, such as skipping RRs, might be required to rescue projects in some circumstances.
6) Increased transactions may impact “gas fees”, which are transaction costs in the public blockchain. DAOs often utilize a public blockchain like Ethereum, which requires gas fees for each transaction to maintain its services on the blockchain. The gas fee fluctuates according to the number of transactions within a certain time window, since the number of transaction times is limited, and the price incentivizes people to optimize timing of token trades. DAO activities can be hindered by such matters since they accrue high costs. Some off-chain solutions are proposed to mitigate such issues. In some solutions, including optimistic rollups and zero-knowledge rollups, security is derived directly from the public blockchain. Other solutions, such as sidechains and plasma chains, create new chains, and their security is distinct from the security of the public blockchain. In this manner, the number of transactions per second increases, and increased transactions do not hinder DAO activities due to a substantial reduction in gas fees.
7) Compatibility of reviewer reputational systems is also a matter of concern. Our proposed system envisions integrating a reputation system for reviewers using the blockchain. However, the reliability of the reputation system is undeveloped and uncertain. Instead of integrating this system, we should create a trusted pool of reviewers and make it as functional as the traditional peer review system first of all.
8) With ABCDEF publishing, which utilizes blockchain technology, there are limitations to how it can address environmental concerns, but there are also potential solutions to mitigate its impact. There are efforts to develop more energy-efficient blockchain algorithms and explore alternative energy sources, such as renewable energy, to power blockchain networks. Additionally, some decentralized publishing platforms are exploring using off-chain solutions to reduce the energy consumption required for transactions. While there are limitations to how ABCDEF publishing can address environmental concerns, there are potential solutions to mitigate its impact, such as developing more energy-efficient algorithms and promoting the reuse and recycling of electronic devices. It is important to continue exploring and implementing these solutions to ensure that the benefits of blockchain technology can be realized while minimizing its negative impact on the environment.
9) Some scientists might insist there are strict regulations and accredited committees to review research with human subjects or animals in many countries. Such committees usually do not process applications before studies are granted. Also, they might think there are huge differences between grant and ethics applications and an introduction plus the method section of a research publication. These criticisms are not strange to researchers accustomed to the current process. However, as we have argued in our earlier paper, ^14^ the process of applying for a grant and then conducting an ethical review may be one factor that has led to ethical deviations. Therefore, ABCDEF proposes the reversal of such a process. Moreover, as for grand and ethical considerations that require more than the background and methods section of the paper, those issues can be addressed by providing information as Supplementary information in the registered report. Of course, our proposed system may require the cooperation of existing IRBs in various situations. Additionally, this system must gain recognition from such institutions IRB and other relevant entities. The arrangement that enables cooperation with the respective institutional ethics review committees and publicity of our proposed system should be considered as issues to be addressed in the future.

## Conclusion

We believe that ABCDEF publishing can effectively solve a panoply of problems facing the scientific community today. At the time of writing, we are just beginning to construct this system, which is community-based and developed on the blockchain. Most importantly, it will be realized as a large-scale movement involving various stakeholders, including scientists, publishers, and citizens.

## Data Availability

OSF: ABCDEF publishing. https://doi.org/10.17605/OSF.IO/NSVZD. ^35^ This project contains the following extended data:
- Supplementary Information.pdf Data are available under the terms of the Creative Commons Zero “No rights reserved” data waiver (CC0 1.0 Public domain dedication).
