# Supplementary material for: Autonomous, bidding, credible, decentralized, ethical, and funded (ABCDEF) publishing
Source: F1000Res. 2023 Oct 5;12:877. Originally published 2023 Jul 25. [Version 2] doi: 10.12688/f1000research.130188.2 (PMC10831225; doi:10.12688/f1000research.130188.2)
Supplement: Peer review history

# Reviewer report: Reviewer response for version 2

**DOI:** 10.5256/f1000research.156966.r233908

**Published:** 2024-01-31

**Authors:** Tae-Sul Seo

**Affiliations:** 1 Korea Institute of Science and Technology Information, Daejeon, South Korea

In the article, the authors proposed a procedure in which three kinds of peer reviews, including a scientific peer review, an ethical review, and a funding review, are executed simultaneously based on Web3 technology. The procedure includes five phases: proposal and review, blockchain voting for funding, research and writing, peer review for the final report, and publishing in a friendly journal. However, the article has several substantial problems below.

 1) It has no novel ideas except to integrate several scholarly steps using blockchain technology. The core concept of the article, performing three types of peer reviews simultaneously, is too ideal. The authors should not insist but provide evidence for the efficiency of the Trinity review than the traditional one.

 2) Besides, blockchain-based peer review systems are not a new approach. We can find several novel articles dealing with blockchain-based ecosystems for publishing. Please see below articles:

 [Ref-1]

 [Ref-2]

 3) The title of the article exaggerates. For example, the article doesn't explain the bidding process. Blockchain technology doesn't guarantee the credibility of research by itself. The title should be 'a combined procedure for research funding review and publishing based on Web3'.

Is the rationale for developing the new method (or application) clearly explained?

Yes

Is the description of the method technically sound?

Yes

Are the conclusions about the method and its performance adequately supported by the findings presented in the article?

Partly

If any results are presented, are all the source data underlying the results available to ensure full reproducibility?

No source data required

Are sufficient details provided to allow replication of the method development and its use by others?

Partly

Reviewer Expertise:

open peer review

I confirm that I have read this submission and believe that I have an appropriate level of expertise to confirm that it is of an acceptable scientific standard, however I have significant reservations, as outlined above.

## References

[1] : Peer Review: Toward a Blockchain-enabled Market-based Ecosystem. *Communications of the Association for Information Systems* .2018;42: 10.17705/1CAIS.04228 646-653 10.17705/1CAIS.04228

[2] : On the prospects of blockchain and distributed ledger technologies for open science and academic publishing. *Semantic Web* .2018;9(5) : 10.3233/SW-180322 545-555 10.3233/SW-180322

# Reviewer report: Reviewer response for version 2

**DOI:** 10.5256/f1000research.156966.r212394

**Published:** 2023-11-28

**Authors:** Timothy Errington

**Affiliations:** 1 Center for Open Science, Charlottesville, Virginia, USA

The authors have addressed all of my concerns.

Is the rationale for developing the new method (or application) clearly explained?

Yes

Is the description of the method technically sound?

Partly

Are the conclusions about the method and its performance adequately supported by the findings presented in the article?

Partly

If any results are presented, are all the source data underlying the results available to ensure full reproducibility?

No source data required

Are sufficient details provided to allow replication of the method development and its use by others?

No

Reviewer Expertise:

NA

I confirm that I have read this submission and believe that I have an appropriate level of expertise to confirm that it is of an acceptable scientific standard.

# Reviewer report: Reviewer response for version 1

**DOI:** 10.5256/f1000research.142922.r199372

**Published:** 2023-09-19

**Authors:** David Mellor

**Affiliations:** 1 Center for Open Science, Charlottesville, Virginia, USA

In "Autonomous, bidding, credible, decentralized, ethical, and funded (ABCDEF) publishing" the authors provide a solution to current systems in scientific research that rely on slow, linear review processes for grants, publication, and ethical review by building on their previous work ("Trinity Reviews") to propose a system that incentivizes participation in the process through blockchain technology. While I believe there are a number of benefits to the proposed system, I also see a number of challenges to overcome that could be addressed in this manuscript. 

 The first, and most substantial shortcoming is the fact that the proposed workflow (Fig 1), will, for most researchers, still require a linear review process, with all of the shortcomings that that entails. In my experience, few researchers submit studies for ethical review unless they are required to do so. The "Institutional ethics review (if necessary)" box is functionally required, and changes by that process are likely to affect the study design, which would require an additional line in the author's figure from that box back up to editorial review after triage. Overcoming that inefficiency should be a more central purpose of this publishing model, and doing that would require working with the ethics review committees in individual universities. In the U.S., they are beholden to government guidelines, and so there could be substantial challenges to integrating them into this process. 

 Another limitation is that the funding review is still, in all cases, separate from the publishing and ethics review. As before, concerns that arise from the funding perspective cannot be addressed without editorial agreement if the "in-principle acceptance" is to be honored (in other words, any changes have to be approved by the editor, who may request additional reviewer insight). 

 If all three sections: editorial, ethics, and funding can be combined, then I would see substantial benefit to this system. Even if the funding section remains seperate (because in this model it is more distributed and focused less on review but rather on up and down votes of being worthy of funding), I still think there is need to integrate institutional ethics review with the scientific review. 

 My final set of comments are minor points that I think can be easily added to this manuscript:

1. What type of funder is this model designed for? Are you proposing that a private research foundation support it, a government agency? It will be important to understand that funder perspective and motivation for participating in order for this model to work. Their considerations (such as funder intent for private foundations, or legal requirements and taxpayer oversight for government agencies) must be taken into account so that there is a chance for them to participate. 
2. In item 7, you note that this is compatible with reviewer systems. Please describe how you would overcome compatibility issues with MULTIPLE systems. Currently, ethics reviews, funder reviews, and article reviews all use different systems, and that is a major barrier to a combined workflow such as you describe. 
3. In item 3, you note a concern about fraudulent accounts. Would it be possible to use simpler verification systems, such as possessing an ORCID and a human reviewer verifying your identity and credentials?

Is the rationale for developing the new method (or application) clearly explained?

Yes

Is the description of the method technically sound?

Partly

Are the conclusions about the method and its performance adequately supported by the findings presented in the article?

No

If any results are presented, are all the source data underlying the results available to ensure full reproducibility?

No source data required

Are sufficient details provided to allow replication of the method development and its use by others?

Partly

Reviewer Expertise:

Open science, meta-science, policy, ecology, citizen science

I confirm that I have read this submission and believe that I have an appropriate level of expertise to confirm that it is of an acceptable scientific standard, however I have significant reservations, as outlined above.

# Response

**Published:** 2023-10-02

**Authors:** Kaito Takashima

**Affiliations:** Kyushu University, Japan

**2-1**. In "Autonomous, bidding, credible, decentralized, ethical, and funded (ABCDEF) publishing" the authors provide a solution to current systems in scientific research that rely on slow, linear review processes for grants, publication, and ethical review by building on their previous work ("Trinity Reviews") to propose a system that incentivizes participation in the process through blockchain technology. While I believe there are a number of benefits to the proposed system, I also see a number of challenges to overcome that could be addressed in this manuscript.

**Reply**: Thank you for your compliments on our proposal. Below, we provide responses to each point that you point out.

**2-2**. The first, and most substantial shortcoming is the fact that the proposed workflow (Fig 1), will, for most researchers, still require a linear review process, with all of the shortcomings that that entails. In my experience, few researchers submit studies for ethical review unless they are required to do so. The "Institutional ethics review (if necessary)" box is functionally required, and changes by that process are likely to affect the study design, which would require an additional line in the author's figure from that box back up to editorial review after triage. Overcoming that inefficiency should be a more central purpose of this publishing model, and doing that would require working with the ethics review committees in individual universities. In the U.S., they are beholden to government guidelines, and so there could be substantial challenges to integrating them into this process.

**Reply**: As you see in the reply to Reviewer 1, we proposed the system as a community for researchers for whom institutional ethics review is not possible in the first place, as we indicate in our previous (Mori et al., 2022) and present papers. As you pointed out, there would be situations where our system needs the cooperation of existing IRBs, and we need to be recognized by the committees. On the other hand, as the previous researchers indicated in the following paper, it is a stark fact that many researchers are unable to conduct research because they do not have their own ethics review board, or because the cost of asking an independent review board is impractical. Your point is important that we have added it as an issue in "Potential weaknesses, threats, and solutions". Simultaneously, we have added the point that proceeding on the premise of the current institutional ethics reviews are themselves an obstacle to broadening the scope of science in the Introduction section:

https://psycnet.apa.org/doiLanding?doi=10.1037%2Fpst0000166

https://explore.bps.org.uk/content/report-guideline/bpsrep.2021.inf180/chapter/bpsrep.2021.inf180.12

 ***Introduction

 "Furthermore, proceeding on the assumption of the current institutional ethical reviews impede broadening the scope of science, considering that many researchers suffer from the lack of opportunities for institutional review."

 ***Potential weaknesses, threats, and solutions- item9

 "Of course, our proposed system may require the cooperation of existing IRBs in various situations. Additionally, this system must gain recognition from such institutions IRB and other relevant entities. The arrangement that enables cooperation with the respective institutional ethics review committees and publicity of our proposed system should be considered as issues to be addressed in the future." 

**2-3**. Another limitation is that the funding review is still, in all cases, separate from the publishing and ethics review. As before, concerns that arise from the funding perspective cannot be addressed without editorial agreement if the "in-principle acceptance" is to be honored (in other words, any changes have to be approved by the editor, who may request additional reviewer insight).

**Reply**: Basically, it is not envisioned that the funding phase will be reverted back to another previous phase after the IPA. If the (funding) voters feel that there are any problems with the research protocol/content at voting, they can simply not vote the project. If the researcher wants to modify the protocol and still receive funding from the system as a result of rejection, they can withdraw and resubmit the manuscript to our system. This part is not considered a limitation, but rather, the review of research funding is an evaluation and voting process, and asking for specific comments on the content and modifications would be very complicated; it may discourage anyone from joining the funding voting.

**2-4**. If all three sections: editorial, ethics, and funding can be combined, then I would see substantial benefit to this system. Even if the funding section remains separate (because in this model it is more distributed and focused less on review but rather on up and down votes of being worthy of funding), I still think there is need to integrate institutional ethics review with the scientific review.

**Reply**: As we replied to your comment above, we do not require institutional review in our framework only, but at the same time we understand that some studies should actually be required.

**2-5**. My final set of comments are minor points that I think can be easily added to this manuscript:

 What type of funder is this model designed for? Are you proposing that a private research foundation support it, a government agency? It will be important to understand that funder perspective and motivation for participating in order for this model to work. Their considerations (such as funder intent for private foundations, or legal requirements and taxpayer oversight for government agencies) must be taken into account so that there is a chance for them to participate.

**Reply**: Thank you for your important remark As for funders, we are assuming private research foundations, government agencies, or investors, as you have indicated. The format of the fund could be in the form of other DAOs, crowdfunding, or, as reviewer 1 gave us previous examples, the system launching a grant project with an open call for proposals. However, this model is not limited to these specific funders or formats, but aims to support research projects with a wide range of funders and diverse funding sources.

**2-6**. In item 7, you note that this is compatible with reviewer systems. Please describe how you would overcome compatibility issues with MULTIPLE systems. Currently, ethics reviews, funder reviews, and article reviews all use different systems, and that is a major barrier to a combined workflow such as you describe.

**Reply**: We apologize for the misleading wording here. We don’t want to say that limitation 7 here is compatible with the traditional peer review system, but rather that we are advocating the creation of a reliable reviewer pool and making it as functional as the existing peer review system. As you pointed out, it is difficult to prepare reviewer pools for each of ethics and grant scientific review, but by working with the existing community and using a unified format for evaluation, we could bring the three types of reviews together. The sentences has been revised to be clear as follows:

 ***Potential weaknesses, threats, and solutions- item9

 "Compatibility of reviewer reputational systems is also a matter of concern. Our proposed system envisions integrating a reputation system for reviewers using the blockchain. However, the reliability of the reputation system is undeveloped and uncertain. Instead of integrating this system, we should create a trusted pool of reviewers and make it as functional as the traditional peer review system"

**2-7**. In item 3, you note a concern about fraudulent accounts. Would it be possible to use simpler verification systems, such as possessing an ORCID and a human reviewer verifying your identity and credentials?

**Reply**: We think it is possible. More to the point, it is possible to automatically create a non-exchangeable ID in the form of a combination of ORCID and soulbound tokens, not by human hands. However, the problem of fraudulent accounts has already been pointed out even with ORCID (Teixeira da Silva, 2021. . https://doi.org/10.1016/j.jemep.2021.100692), and tying them together may not solve the problem. Rather, we suggest that it would be more realistic to establish a more reliable ID by linking researcher information and research history with a decentralized token such as the one proposed in our manuscript. However, it might lower the overall risk if it can be tied to not only ORCID but also other accounts e.g., google accounts, github accounts, and so on. We add the combined method to prevent fraudulent accounts as follows:

 ***Potential weaknesses, threats, and solutions- item9

 "Moreover, it might lower the overall risk if it can be tied to other accounts (e.g., ORCID, google accounts, github accounts). "

**References**

 Teixeira da Silva, J. A. (2021). Non-compliance with ethical rules caused by misuse of ORCID accounts: Implications for medical publications in the COVID-19 era. Ethics, Medicine, and Public Health, 18, 100692.

# Reviewer report: Reviewer response for version 1

**DOI:** 10.5256/f1000research.142922.r193287

**Published:** 2023-08-31

**Authors:** Timothy Errington

**Affiliations:** 1 Center for Open Science, Charlottesville, Virginia, USA

This manuscript describes a vision of an alternative publishing process that would combine various emerging features being deployed to decrease bias and increase efficiency using a decentralized approach. Below is a list of recommendations for the author to consider.

 In the introduction, why do the authors say “Accordingly, much research effort is wasted.”. This point is on ethical reviews. Are the authors suggesting there is waste because of having to review modified ethics protocols due to unforeseen changes? That doesn’t seem like waste per se, it’s important to revisit ethical protocols when deviations occur. And the unforeseen aspect doesn’t seem like waste because it was unknown during the initial protocol. I think the authors are trying to say there is research waste because those unforeseen changes by the authors might have been caught if methodological expertise was done alongside the ethical review in the first place. I’d suggest rewriting this to make that clearer. As a note, I also am unsure if I see that as waste – more inefficiency in a system.

 In this model it assumes research progresses in single study/experimental settings, but most research papers currently are a collection of multiple studies/experiments. How will that work in this system? For example, Registered Reports can be submitted with pilot data – or the Registered Report study might be a replication of previous research. How does that work in this system?

 For Phase 1, section B – this does not seem to align with how most research is done. The ethical reviews being independent of the institutional ethical review committees seems like it is adding an additional layer of review. In most journals that implement Registered Reports it is an expectation that the ethical review has already occurred, so approval is in place if the study is given IPA. Some research even requires initial ethical review at the grant review stage. I agree that having ethical and scientific review running in parallel is a good idea since these two areas are different and can influence each other, but if I’m understanding the diagram correctly, it seems like a lot of review might go into the process before it reaches the institutional review committee, which would only add additional revisions of the scientific peer review if ethical considerations need to change. Why not have the institutional ethics review running concurrently with the scientific review opposed to this institutional independent ethics review?

 Related to this, in the ‘Advantages and opportunities’ section that this approach would help researchers who do not have institutional affiliations or institutional ethics committees. There already exists independent ethic review boards (e.g., BRANY) to help with this situation, which are similar to the institutional ethics reviews, how would this be integrated?

 For Phase 2, funding – have the authors considered different workflows for having the funding and protocol peer review done? There are pilots experimenting with having a Registered Report reviewed by funders and journals. Here is a feasibility study of different models ( https://doi.org/10.12688/wellcomeopenres.17028.1) and here is a journal consortium model being implemented ( https://www.cos.io/consciousness).

 Finally, I think the article format is not appropriate for this article. This reads more like an opinion article ( https://f1000research.com/for-authors/article-guidelines/opinion-articles) than a methods article ( https://f1000research.com/for-authors/article-guidelines/method-articles). For example, the last sentence of the paper makes quite a strong statement (i.e., ‘it will be realized’) without any evidence – but the authors are of course open to having that opinion of what they believe could happen (e.g., ‘we believe it could be realized’).

Is the rationale for developing the new method (or application) clearly explained?

Yes

Is the description of the method technically sound?

Partly

Are the conclusions about the method and its performance adequately supported by the findings presented in the article?

Partly

If any results are presented, are all the source data underlying the results available to ensure full reproducibility?

No source data required

Are sufficient details provided to allow replication of the method development and its use by others?

No

Reviewer Expertise:

NA

I confirm that I have read this submission and believe that I have an appropriate level of expertise to confirm that it is of an acceptable scientific standard, however I have significant reservations, as outlined above.

## References

[1] : Evaluating Registered Reports Funding Partnerships: a feasibility study. *Wellcome Open Research* .2021;6: 10.12688/wellcomeopenres.17028.1 10.12688/wellcomeopenres.17028.1 34957336 PMC8672223

# Response

**Published:** 2023-10-06

**Authors:** Kaito Takashima

**Affiliations:** Kyushu University, Japan

**1-1.** This manuscript describes a vision of an alternative publishing process that would combine various emerging features being deployed to decrease bias and increase efficiency using a decentralized approach. Below is a list of recommendations for the author to consider.

**Reply**: Thank you for the important insights. Below, we provide responses to each recommendation or question.

**1-2**. In the introduction, why do the authors say “Accordingly, much research effort is wasted.”. This point is on ethical reviews. Are the authors suggesting there is waste because of having to review modified ethics protocols due to unforeseen changes? That doesn’t seem like waste per se, it’s important to revisit ethical protocols when deviations occur. And the unforeseen aspect doesn’t seem like waste because it was unknown during the initial protocol. I think the authors are trying to say there is research waste because those unforeseen changes by the authors might have been caught if methodological expertise was done alongside the ethical review in the first place. I’d suggest rewriting this to make that clearer. As a note, I also am unsure if I see that as waste – more inefficiency in a system.

**Reply**: As you pointed out, the explanation was misleading. We revised it as follows:

 ***Introduction

 "Moreover, approved proposals must often be revised due to unforeseen changes in experimental methodology, necessitating reassessment by post hoc ethics or peer review.5 This rework could be prevented if methodological expertise review is done alongside the ethical review. "

**1-3**. In this model it assumes research progresses in single study/experimental settings, but most research papers currently are a collection of multiple studies/experiments. How will that work in this system? For example, Registered Reports can be submitted with pilot data – or the Registered Report study might be a replication of previous research. How does that work in this system?

**Reply**: Thank you for pointing this out. We apologize for the lack of explanation. We consider this system to be based on a Registered Report (RR) as we described in the manuscript. Therefore, the system naturally accepts such cases as you have suggested. We clearly explain in the manuscript that we accept such cases in the Advantages and opportunities as follows:

 Advantages and opportunities

 ***"In addition, as seen in previous RRs, the ABCDEF system will accept submissions along with pilot data, replications of previous studies, and collections of various experiments and studies, allowing each content to be tied to the blockchain for reference."

**1-4**. For Phase 1, section B – this does not seem to align with how most research is done. The ethical reviews being independent of the institutional ethical review committees seems like it is adding an additional layer of review. In most journals that implement Registered Reports it is an expectation that the ethical review has already occurred, so approval is in place if the study is given IPA. Some research even requires initial ethical review at the grant review stage. I agree that having ethical and scientific review running in parallel is a good idea since these two areas are different and can influence each other, but if I’m understanding the diagram correctly, it seems like a lot of review might go into the process before it reaches the institutional review committee, which would only add additional revisions of the scientific peer review if ethical considerations need to change. Why not have the institutional ethics review running concurrently with the scientific review opposed to this institutional independent ethics review?

**Reply**: We apologize the position of "Ethical Review at the Institution" in the figure is confusing. In our proposed framework, undergoing ethical review at the institution was considered a special case. This primarily occurs when an ethics review is conducted simultaneously with the initial scientific review, and it is recommended that reviewers cannot make a clear judgment, or it is recommended to undergo an institutional ethics review as well. Therefore, in line with the original concept, it is not in its current position but should be included within the Phase 1 Combined Review and the revision loop. We have modified Figure 1 to align with the original intention.

**1-5**. Related to this, in the ‘Advantages and opportunities’ section that this approach would help researchers who do not have institutional affiliations or institutional ethics committees. There already exists independent ethic review boards (e.g., BRANY) to help with this situation, which are similar to the institutional ethics reviews, how would this be integrated?

**Reply**: Indeed, there is already an independent ethics review committee for those who do not have an ethics review committee within their affiliation. However, as pointed out in the background of our manuscript, the researchers must submit documents that are almost identical in content to those submitted for academic peer review in Stage 1 for even such institutions in their own format. When considering this structural and inefficient problem, our proposal system could exclude the barrier to undergoing an ethics review. Also, we’d appreciate it if you check our reply to Reviewer 2. We add the related sentence in the section as follows:

 ***Advantages and opportunities

 "Although there is already an independent ethics review committee for such researchers, they must submit documents that are almost identical in content to those submitted for academic peer review in Stage 1 in their format. Our proposal system could exclude the barrier by integrating all stages."

**1-6**. For Phase 2, funding – have the authors considered different workflows for having the funding and protocol peer review done? There are pilots experimenting with having a Registered Report reviewed by funders and journals. Here is a feasibility study of different models ( https://doi.org/10.12688/wellcomeopenres.17028.1) and here is a journal consortium model being implemented ( https://www.cos.io/consciousness).

**Reply**: Thank you for sharing this very innovative and important precedent! The direction that journals and funders, such as the ones you mentioned, are taking in setting up grant projects and having manuscripts submitted to them is a very compatible. For your question, we have not considered the sperate flows between funding and RR peer review because it might waste the time of researchers. This is one of our motivation to propose the system. However, the precedential funding system which you introduced can be applied in our case with necessary modification. We added the possibility of combination in the Phase 5 explanation as follows:

 “Another approach to grants involves journals of funding organizations initiating grant projects and allowing the submission of manuscript to them (Clark et al. 2021; Center for Open Science)” 

**1-7**. Finally, I think the article format is not appropriate for this article. This reads more like an opinion article ( https://f1000research.com/for-authors/article-guidelines/opinion-articles) than a methods article ( https://f1000research.com/for-authors/article-guidelines/method-articles). For example, the last sentence of the paper makes quite a strong statement (i.e., ‘it will be realized’) without any evidence – but the authors are of course open to having that opinion of what they believe could happen (e.g., ‘we believe it could be realized’).

**Reply**: We initially submitted the article as an Opinion article, but changed it to Methods article after consulting with the Editor on the submission. While we agree with your opinion, we would like to leave the final decision to the Editor.

**References**

 Clark, R., Drax, K., Chambers, C. D., Munafò, M., & Thompson, J. (2021). Evaluating Registered Reports Funding Partnerships: a feasibility study. Wellcome Open Research, 6, 231. https://doi.org/10.12688/wellcomeopenres.17028.1
